# Dynamic modules of the coactivator SAGA in eukaryotic transcription

**DOI:** 10.1038/s12276-020-0463-4

**Published:** 2020-07-03

**Authors:** Youngseo Cheon, Harim Kim, Kyubin Park, Minhoo Kim, Daeyoup Lee

**Affiliations:** 1 Department of Biological Sciences, Korea Advanced Institute of Science and Technology, Daejeon, 34141 South Korea; 2 Leonard Davis School of Gerontology, University of Southern California, Los Angeles, CA 90089 USA

**Keywords:** Transcriptional regulatory elements, Gene regulation

## Abstract

SAGA (Spt-Ada-Gcn5 acetyltransferase) is a highly conserved transcriptional coactivator that consists of four functionally independent modules. Its two distinct enzymatic activities, histone acetylation and deubiquitylation, establish specific epigenetic patterns on chromatin and thereby regulate gene expression. Whereas earlier studies emphasized the importance of SAGA in regulating global transcription, more recent reports have indicated that SAGA is involved in other aspects of gene expression and thus plays a more comprehensive role in regulating the overall process. Here, we discuss recent structural and functional studies of each SAGA module and compare the subunit compositions of SAGA with related complexes in yeast and metazoans. We discuss the regulatory role of the SAGA deubiquitylating module (DUBm) in mRNA surveillance and export, and in transcription initiation and elongation. The findings suggest that SAGA plays numerous roles in multiple stages of transcription. Further, we describe how SAGA is related to human disease. Overall, in this report, we illustrate the newly revealed understanding of SAGA in transcription regulation and disease implications for fine-tuning gene expression.

## Gene activation: many modules make light work

A protein that helps add epigenetic information to genome, SAGA, controls many aspects of gene activation, potentially making it a target for cancer therapies. To fit inside the tiny cell nucleus, the genome is tightly packaged, and genes must be unpacked before they can be activated. Known to be important in genome opening, SAGA has now been shown to also play many roles in gene activation. Daeyoup Lee at the KAIST, Daejeon, South Korea, and co-workers have reviewed recent discoveries about SAGA’s structure, function, and roles in disease. They report that SAGA’s complex (19 subunits organized into four modules) allows it to play so many roles, genome opening, initiating transcription, and efficiently exporting mRNAs. Its master role means that malfunction of SAGA may be linked to many diseases.

## Introduction

Eukaryotic gene expression is extensively regulated, mostly at the transcription initiation step. Nucleosomes bind to promoters and form a physical barrier that blocks transcription, indicating that a destabilizing interaction between DNA and histones is important for active transcription. As acetylation of lysine residues in the histone tail region neutralizes the positive charge of histones and diminishes the electrostatic interaction between histones and the negatively charged phosphodiester backbone of DNA, histone acetylation has long been considered to be generally required for active transcription^1^ and other biological processes that demand DNA access^2^.

SAGA (Spt-Ada-Gcn5 acetyltransferase) is a eukaryotic transcription coactivator complex that controls transcription by modifying histones. The story of SAGA started with the discovery of that the transcription coactivator Gcn5 was a nuclear histone acetyltransferase (HAT)^3^. Although Gcn5p showed HAT activity with free histones, it did not acetylate nucleosomal histones; this finding led to the discovery of functional HAT complexes containing Gcn5p^4^. Two HAT complexes were found in yeast: the 1.8-MDa SAGA complex and the 0.8-MDa ADA complex.

HAT complexes containing the Gcn5p homolog were also found in humans and were independently named the SPT3-TAF9-GCN5 acetyltransferase (STAGA) complex and TATA-binding protein-free TAF-containing complex (TFTC)^5,6^. STAGA and TFTC were initially regarded as distinct complexes, but more recently, they both have been increasingly recognized as corresponding to the human SAGA (hSAGA) complex. SAGA has retained its transcriptional coactivator function throughout evolution, although its specific role in transcription has been modified in some species. For example, yeast SAGA participates in the transcription of stress-inducible genes, such as heat-shock genes^7–10^, whereas hSAGA activity has been observed at ER stress-induced promoters but does not appear to be involved in the p38 MAPK pathway-mediated acetylation of histones at sodium arsenite-induced promoters^11^. In addition to the classical coactivator function of its HAT activity, SAGA regulates transcription via Ubp8p, which is a ubiquitin-specific protease (UBP) that catalyzes H2Bub1 deubiquitylation^12–15^. Multiple lines of evidence developed over the past decade have shown that the SAGA subcomplex critical for this DUB activity also regulates other aspects of gene expression, including the nucleocytoplasmic export of mRNAs^16,17^. Collectively, these studies suggest that the SAGA complex may comprehensively coordinate the entire gene expression process and that, conversely, malfunction of SAGA may deregulate gene expression in a manner that may be linked to various diseases.

Before presenting details, we declare that, in this review, the proteins discussed are designated by the standard nomenclature for the corresponding organism. For example, the Gcn5 protein in *Saccharomyces cerevisiae* is designated “Gcn5p”; in *Schizosaccharomyces pombe*, it is “Gcn5”; and in *Homo sapiens*, it is “GCN5.” The nomenclature of other SAGA subunits in four representative model organisms is summarized in Table 1^18,19^.

**Table 1:** Subunits of SAGA in four representative model organisms.

Module	*Saccharomyces cerevisiae*	*Schizosaccharomyces pombe*	*Drosophila melanogaster*	*Homo sapiens*
HAT module	Gcn5p	Gcn5	dKAT2 (dGcn5)	KAT2A (GCN5) /KAT2B (PCAF)
	Ada2p	Ada2	dAda2b	TADA2b
	Ada3p (Ngg1p)	Ada3 (Ngg1)	dAda3	TADA3
	Sgf29p	Sgf29	Sgf29	SGF29 (CCDC101)
Core module	Taf5p	Taf5	Wda	TAF5L (PAF65β)
	Taf6p	Taf6	Saf6	TAF6L (PAF65α)
	Taf9p	Taf9	dE(y)1 (Taf9)	TAF9/TAF9b
	Taf10p	Taf10	Taf10b	TAF10 (STAF28)
	Taf12p	Taf12	Taf12	TAF12
	Ada1p	Ada1	Ada1	TADA1 (STAF42)
	Spt7p	Spt7	dSpt7	SUPT7L (STAF65γ)
	Spt20p	Spt20	Spt20	SUPT20H
	Spt3p	Spt3	dSpt3	SUPT3H
	Spt8p	Spt8	–	–
TF-binding module	Tra1p	Tra1	Nipped-A (dTra1)	TRRAP
DUB module	Ubp8p	Ubp8	dNonstop	USP22 (UBP22)
	Sgf11p	Sgf11	dSgf11	ATXN7L3
	Sgf73p	Sgf73	dATXN7	ATXN7 (SCA7)
	Sus1p	Sus1	dE(y)2	ENY2
Splicing module	–	–	Sf3b3	SF3B3
	–	–	Sf3b5	SF3B5

## Functional modules of SAGA

**Fig. 1:**
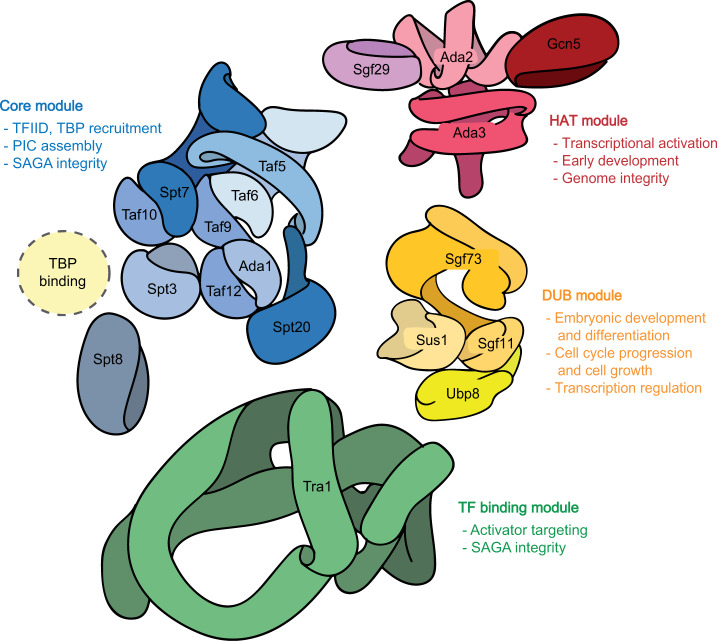
Schematic diagram showing the modular organization of the SAGA complex. For the sake of simplicity, each subunit is labeled with the name used in *S. cerevisiae*. The schematic diagram shows the major functions of each module and presents recent structural data obtained from yeast^49,53,64^. Subunits belonging to each module are colored similarly: red, HAT module; blue, core module; green, TF-binding module; and yellow, DUB module. Subunits having a histone octamer-like fold in the core module are depicted as half circles that form circles with their corresponding partners: Taf6-Taf9; Ada1-Taf12; Spt7-Taf10; and Spt3, which has two octamer-like folds. The dotted circle near Spt3 and Spt8 indicates the TBP-binding site, where TBP is recruited at the transcription initiation step.

Yeast SAGA is composed of 19 subunits that are organized into 4 functionally distinct modules: the HAT module (Gcn5p, Ada2p, Ada3p, and Sgf29p), the core structural module (Taf5p, Taf6p, Taf9p, Taf10p, Taf12p, Ada1p, Spt7p, Spt20p, Spt3p, and Spt8p), the DUB module (Ubp8p, Sgf73p, Sus1p, and Sgf11p), and the transcription factor-binding (TF-binding) module (Tra1p)^18,19^ (Fig. 1 and Table 1). The modular organization of SAGA was first revealed by genetic studies in yeast^20^ and was subsequently supported by biochemical experiments and electron microscopy^21^. In higher eukaryotes, the splicing module (SF3B3 and SF3B5) was also found as a subcomplex of the SAGA complex^22–24^. The splicing module facilitates the activation and proper splicing of some SAGA-regulated transcripts, but the specific role of the SF3B subcomplex within SAGA warrants further investigation^24^. The compartmentalization of these functional modules allows the SAGA complex to intricately and dynamically regulate gene expression, as discussed in detail below.

### HAT module

**Fig. 2:**
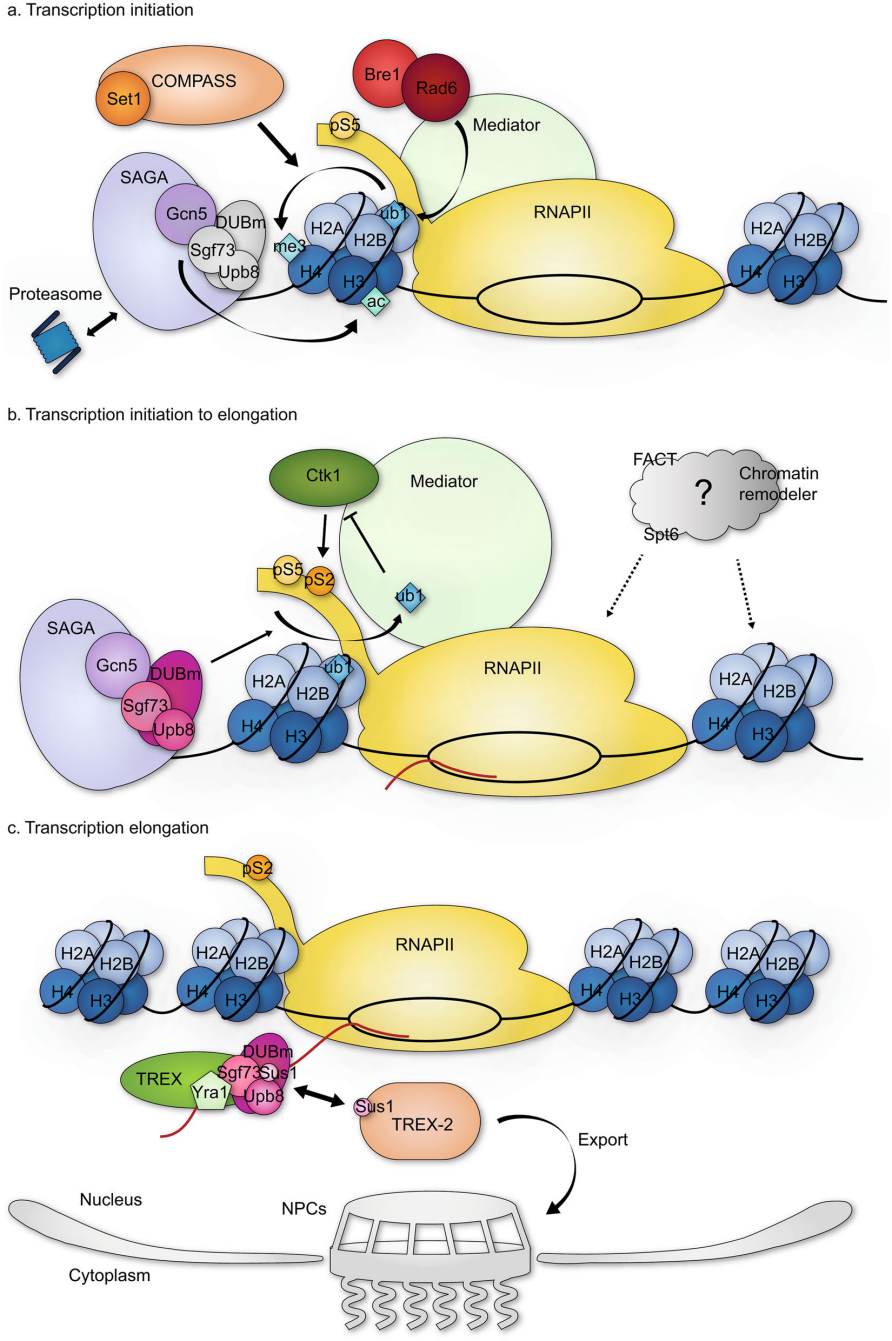
SAGA regulates transcription to fine-tune gene expression. Subunits that are major in each step are colored; otherwise, they are uncolored. **a** SAGA promotes an open chromatin structure through its HAT activity, which can be allosterically regulated by the proteasome to favor transcription initiation^67,110^. When this occurs, Bre1/Rad6-dependent H2Bub1 triggers the methylation of histone H3 by Set1, yielding TSS-associated histone modifications that act as markers to recruit downstream effectors that facilitate transcription initiation. **b** Deubiquitylation of ubiquitylated H2B is mediated by DUBm and is necessary for the recruitment of Ctk1, which phosphorylates Ser2 of RNAPII CTD and allows the release of paused RNAPII^104^. For productive elongation, the nucleosome barrier must be overcome. Histone chaperones (FACT and Spt6) and chromatin remodelers may be essential in this process and may cooperatively regulate the transition from initiation to transcription elongation. **c** The Rpt2p-Sgf73p interaction leads to the separation of DUBm, and the separated Sgf73 contributes to cotranscriptional mRNP surveillance and the mRNA export pathway^93,111^. Sus1, which is a subunit of both DUBm and TREX-2, may mediate the targeting of genes to nuclear pore complexes (NPCs).

Gcn5p, which is one of the best-studied proteins of the GCN5-related N-acetyltransferase (GNAT) superfamily, serves as the catalytic subunit of the HAT module (Fig. 2a). Recombinant Gcn5p acetylates free histones but is not sufficient to acetylate nucleosomal histones alone^25^. Biochemical and structural studies have revealed that Ada2p potentiates Gcn5p HAT activity by cooperatively binding to Gcn5p and changing its conformation to a catalytically active form. Specifically, Ada2p promotes Gcn5p binding to acetyl-CoA^26^. Remarkably, yeast with *ADA2* deletion showed decreased telomeric silencing, indicating that Ada2p plays a specific role in maintaining genomic stability^27^. Deletion of *GCN5* did not yield a comparable phenotype^27^, implying that Ada2p participates in telomeric silencing through its own targeting activity, independent of the HAT activity of Gcn5p.

Humans have two paralogs of the yeast Gcn5p protein, GCN5 and p300/CBP-associated factor (PCAF)^28^. PCAF is found in both the SAGA complex and p300/CBP, where it contributes to the activation of transcription. The most significant difference between GCN5 and PCAF is the E3 ubiquitin ligase domain of the latter, which may enable PCAF to regulate the stability of transcription factors or signaling proteins and thereby participate in diverse regulatory mechanisms^29,30^.

The HAT module is shared between the SAGA and ADA complexes in *S. cerevisiae*^4,31^. The ADA complex consists of the SAGA HAT module and two additional subunits, Ahc1p and Ahc2p^32^. Although the ADA complex lacks an activator-targeting subunit, it is thought to be recruited to chromatin with relatively low specificity through the bromodomain of Gcn5p or the activator-domain-binding sites of Ada2p/Ada3p, whereby it helps maintain the global histone acetylation level. A recent study suggested that there may be an ADA-equivalent complex in *Drosophila*^33^; however, no homolog subunit of Ahc1p or Ahc2p has yet been identified in this model. Thus, it is unclear whether this proposed complex can be considered homologous to the yeast ADA complex or whether it is simply a different form of the SAGA HAT module.

In metazoans, the HAT module is also found in the Ada2a-containing (ATAC) complex^34–36^, although the composition of the ATAC HAT module differs slightly from that of the SAGA HAT module. Metazoans have two paralogous ADA2 proteins, ADA2a and ADA2b, which are specific to the ATAC HAT module and SAGA HAT module, respectively^37,38^. They also differ in that ATAC possesses a second HAT subcomplex, ATAC2, which is conserved in flies and mammals^39,40^. Differences in catalytic subunits and adapter protein species direct distinct HAT activities: histone H3-specific acetyltransferase activity is observed with SAGA, whereas H3/H4-specific activity is observed with ATAC^39,41,42^. Other subunits of the ATAC complex (YEATS2, ATAC1/ZZZ3, MBIP, WDR5, and DR1/NC2β) have been suggested to be involved in structural or regulatory functions; a recent study partially supported this idea by showing that ATAC1 modulates the histone H3 acetyltransferase activity of the ATAC complex^43^.

ChIP-seq data for ATAC and SAGA showed that these two HAT complexes possess overlapping and nonoverlapping binding sites^44^. While SAGA prefers promoters to enhancers^45^, ATAC is associated with both enhancers and promoters. The following are among the yet-unanswered questions: which proteins are responsible for targeting ATAC specifically on enhancers, and which ATAC subunits interact with them?

### Core module

The core structural module, which is the largest module in the SAGA complex, consists of ten subunits. It critically contributes to the assembly of the preinitiation complex (PIC) by recruiting TBP and transmitting signals from the TF-binding module to the HAT and DUB modules. Taf6p-Taf9p, Taf10p-Spt7p, Taf12p-Ada1p, and Spt3p have histone-like fold domains (HFDs) and together form an asymmetric octamer-like fold^46^ (Fig. 1). In yeast, five subunits containing HFDs are shared between the core module of SAGA and the general TF complex of TFIID: Taf5p, Taf6p, Taf9p, Taf10p, and Taf12p. In *Drosophila* and humans, however, Taf5 and Taf6 are not in TFIID; instead, TAF5L and TAF6L are preferentially found in SAGA^47^, while TAF5 and TAF6 are in TFIID. The relative positions of the corresponding TAFs are conserved between SAGA and TFIID, including the TBP-binding site in these complexes and the histone octamer-like folds. These TAFs, shared between SAGA and TFIID, are thought to be important for promoting PIC formation by SAGA.

In yeast, Ada1p, Spt7p, and Spt20p are integral to the proper assembly of the SAGA complex^4,48^. A structural analysis of the SAGA core module organization showed that Taf5p is central for core module assembly and provides docking sites for histone fold pairs on its C-terminal WD40 domain^49^. Spt20p was found to critically stabilize the complex by providing a wedge-like structure that can be intercalated between the WD40 domain and N-terminal domain of Taf5p. Notably, a recent study on *S. pombe* showed that *SPT20* deletion did not affect the assembly of SAGA complex subunits, except for DUBm and Tra1^50^. These results suggest that, at least in *S. pombe*, Spt20 is not necessary for SAGA assembly.

As characterized by biochemical assays and supported by structural data, Spt3p and Spt8p are known to bind TBP, load it onto a promoter, and promote the assembly of the PIC. For genes that are inhibited by SAGA, however (e.g., *HIS3* and *TRP3*), Spt3p and Spt8p may prevent the binding of TBP to the TATA box^51^. Although Spt3p is conserved in Drosophila and humans, no homolog of Spt8p has been found in metazoans. The null mutation of Spt3p results in mating-defective phenotypes, sporulation in diploids, and invasive growth in haploids; in contrast, the *SPT8*-null mutant shows no severe defect, suggesting that the loss of Spt8p during evolution would not have had a significant effect. In this regard, metazoan SAGA can be compared with the yeast SAGA-like (SLIK) complex, which lacks Spt8p due to truncation of the Spt8p-interacting helices in Spt7p^52^. Uncovering the detailed roles of the SLIK complex might improve our understanding of how SAGA has changed throughout evolution.

Recent cryogenic electron microscopy (cryo-EM) images showed that the C-terminal stirrup region of TBP is bound by the Spt3p pocket, whereas the N-terminal region of TBP is in contact with Spt8p^53^. Consistent with early biochemical evidence, the *SPT8*-null mutant does not induce any alteration in the physical interaction between Spt3p and TBP^54^. Together, these findings suggest that Spt3p is a major player in the binding of TBP, whereas Spt8p tends to play auxiliary and/or regulatory roles.

### TF-binding module

The TF-binding module comprises a single protein, Tra1p, and forms the largest subunit (~433 kDa) of SAGA. Tra1p belongs to the phosphoinositide 3 kinase-related kinase (PIKK) family but lacks catalytic activity, making it the only pseudokinase of the PIKK family^55,56^. Similar to other PIKK family proteins, Tra1p requires chaperone and cochaperone proteins to ensure that it is properly folded and assembled on the HAT complex^50,57,58^. Tra1p is composed of three domains: the HEAT (Huntingtin, elongation factor 3, PR65/A, and TOR) domain, which harbors various binding sites for acidic activators, including Gcn4p, Gal4p, and Rap1p; the FAT (FRAP, ATM, and TRRAP) domain; and the PIKK domain^59,60^. Recent structural data suggested that Tra1p binds to the SAGA core module using a cup-shaped motif in the FAT domain^50^. A narrow hinge formed between Tra1 and the core module is thought to provide structural flexibility for regulatory functions^60^.

Tra1p is also found in the NuA4 HAT complex^61^ as part of the activator-targeting module of NuA4^62^. The only exception to this pattern is found in *S. pombe*, where two paralogous genes correspond to *TRA1*; designated *TRA1* and *TRA2*, these proteins are exclusively found in SAGA and NuA4, respectively^63^. The nonoverlapping distribution of Tra1 and Tra2 provides a way to characterize the SAGA- or NuA4-specific roles of Tra1p. In contrast to SAGA, which has other subunits that can bind TFs, none of the NuA4 subunits is known to interact with TFs. Considering that Tra1-depleted cells are viable but Tra2-depleted cells are not, the necessity of Tra1 for the viability of other organisms likely reflects the importance of the appropriate targeting of NuA4.

Since SAGA and NuA4 from *S. cerevisiae* occupy distinct facets of Tra1p, the access of activators to the Tra1p-activator-binding domains would be restricted depending on the HAT complex to which Tra1p is bound^64^. For instance, Tra1p and Eaf1p form the structural core of NuA4^65^, whereas Tra1p is at periphery of the SAGA complex^64^, where its integrity is maintained by the core subunit. Thus, because distinct facets of Tra1p interact with HAT complexes, Tra1p might recognize different populations of TFs. A recent study on SAGA and NuA4 showed that depletion of the HAT catalytic subunits of SAGA and NuA4 led to different effects on Pol II occupancy^66^, implying that their targeting might be distinct. Nucleosome binding induces a structural change in the SAGA complex^49^ in a manner that displaces the HAT module and the DUB module from the core module, enabling them to bind to the nucleosome in the proper orientation. Considering that an activator is generally thought to be important in determining whether the coactivator binds a genomic region (and the associated nucleosomes), one possible supposition is that Tra1 may induce a conformational change in SAGA upon binding to activator. Studies have shown that nucleosomal HAT activity is more efficient when the HAT module is in a separate ADA complex than when ADA is incorporated into SAGA^31^, suggesting that allowing the HAT module to become more flexible upon TF binding may enable the precise regulation of the nucleosomal HAT activity of SAGA.

### DUB module

Histone H2B ubiquitylation has been shown to be essential for many chromatin-based actions, but its role in transcription regulation is undoubtedly the most thoroughly studied. Works from many laboratories have revealed the existence of cotranscriptional H2B ubiquitylation–deubiquitylation cycles and their roles in directly stimulating transcription elongation^67^. The SAGA DUB module (DUBm) in *S. cerevisiae* consists of the catalytic Ubp8p (ubiquitin protease 8) subunit along with Sgf11p (SAGA-associated factor 11), Sus1p (SI gene upstream of ySa1), and Sgf73p (SAGA-associated factor 73). The deubiquitylation of H2Bub1 by SAGA was first discovered by researchers studying *S. cerevisiae* Ubp8p; they demonstrated that both H2B ubiquitylation and its successive deubiquitylation are essential for gene activation^14,15^. A second component of DUBm, Sgf11p, was subsequently identified and found to be required for the deubiquitylation process^68–70^. Next, Sus1p was identified as a component of both SAGA and the Sac3p-Thp1p mRNA export complex^71^, which was later named TREX-2. Sus1p, whose recruitment to SAGA depends on Ubp8p and Sgf11p, has also been shown to be required for the H2B-deubiquitylation activity of DUBm^72^. Sgf73p, which was initially identified as a novel component of SAGA through a proteomic approach^73^, is required for the proper assembly of DUBm onto the SAGA complex and the recruitment of TREX-2 to SAGA. A physical interaction between SAGA and TREX-2 is essential for targeting the transcriptional machinery to the periphery of the nuclear pore complex (NPC) in a phenomenon known as “gene gating”^74,75^.

Studies have shown that Ubp8p alone cannot carry out the deubiquitylation reaction^69^, and its zinc-finger (ZnF)-UBP domain cannot bind to free ubiquitin^68^, suggesting that Ubp8p activity may be subject to allosteric regulation promoted by a nonsubstrate partner (likely another DUBm subunit). The minimal DUBm structure that confers full deubiquitylation of the nucleosome consists of Ubp8p, Sgf11p, Sus1p, and an N-terminal fragment of Sgf73p (amino acids 1–104)^74^. Several studies have shed light on the structural basis for the deubiquitylation activity of DUBm^76–78^. Sgf11p was found to activate Ubp8p through its C-terminal ZnF domain, which directly interacts with the C-terminal catalytic domain of Ubp8p. An interaction between Sus1p and the helix of Sgf11p and between Sus1p and the ZnF domain of Ubp8p yields an entirely closed conformation of DUBm that seems to stabilize its activity. Sgf73p facilitates the coupling of the “catalytic lobe” and the “assembly lobe” of DUBm, which act together as a unified module through its C-terminal fragment, which is required to connect DUBm with the remainder of SAGA^53,79^. Overall, the four subunits seem to have a highly intertwined arrangement. Recent structural studies elucidated the chromatin-binding interface of DUBm^80^, and these findings suggested that there are interactions between the ZnF domain of Sgf11p and the conserved acidic patch in histone H2A/H2B^81^ and between the catalytic domain of Ubp8p and histone H2B. However, the crystal structure assessed in the abovementioned reports did not include the SCA7 domain within Sgf73p, which contains the second ZnF domain and was previously reported as a nucleosome-binding domain^82^. Notably, a recent in vitro study suggested that a fine-tuned regulatory mechanism for generating sophisticated ubiquitylation patterns might involve the competition of DUBm with Bre1p for binding the acidic patch in the nuclear core particle^83^.

DUBm is clearly conserved in *Drosophila melanogaster*^84,85,86^ and *H. sapiens*^87–89^. In contrast, the *ATXN7* (the human ortholog of yeast Sgf73p) gene has undergone diversification during evolution and is found as two paralogs, *ATXN7L1* and *ATXN7L2*, in *Mus musculus* and *H. sapiens*^86,87,90^. Both paralogs have been described as being part of the SAGA complex, as assessed through proteomic analysis^91^. Another subunit of DUBm, *ATXN7L3* (the human ortholog of yeast Sgf11p), also has a paralog called *ATXN7L3B*. However, ATXN7L3B localizes to the cytoplasm and does not associate with either SAGA or TREX-2, although it interacts with ENY2 (the human ortholog of yeast Sus1p)^92^.

DUBm has been shown to dissociate from SAGA under certain conditions. In *S. cerevisiae*, the 19S regulatory particle (19S RP) of the proteasome facilitates the separation of functional DUBm from SAGA through a physical interaction^93^. Consistent with this finding, studies from different laboratories have shown that DUBm can function as an independent module. For example, it reportedly remained stable following the loss of dAtxn7 (the *Drosophila* ortholog of yeast Sgf73p, which has been reported to structurally connect DUBm with SAGA) in *D. melanogaster*^86^ and upon knockdown of SUPT20H (human ortholog of yeast Spt20p, which is essential for maintaining the integrity of SAGA) in *H. sapiens*^11^. In striking contrast to the effect seen in yeast, a few studies showed that H2B ubiquitylation decreased following the loss of dAtxn7 and ATXN7L3 in metazoans^86,87,90^. Thus, DUBm may maintain its integrity without SAGA or even lose its initial role as a ubiquitin protease and thus, likely has an alternative, its SAGA-independent function.

### The SAGA DUBm and transcription activation and elongation

Histone H2B ubiquitylation facilitates transcription elongation by cooperating with FACT to support efficient nucleosome reassembly^94–96^. H2B ubiquitylation may also directly regulate transcription elongation by changing the chromatin structure. Although the role of H2B ubiquitylation in nucleosome stabilization seems to suggest the opposite effect^97,98^, several biochemical studies have shown that H2B ubiquitylation disrupts chromatin compaction and promotes an open chromatin structure^99–101^. H2B deubiquitylation has also been shown to have a profound effect on subsequent H3 methylation and productive transcription^14,15,88,89,102,103^. Notably, Ubp8p and Rad6p/Bre1p may interact with elongating RNA polymerase II, travel along with it into the coding regions^14,104^, and act predominantly on gene bodies^45^. These findings collectively suggest that repeated and transient cycles of H2B ubiquitylation and deubiquitylation occur in the wake of RNA polymerase II during transcription elongation and function as a key checkpoint for optimal gene activation.

During the early stage of transcription, the PAF (polymerase-associated factor) complex is recruited to RNA polymerase II, with which it interacts through pSer5 in the RNA polymerase II CTD (C-terminal domain). This PAF recruitment facilitates Rad6p/Bre1p-mediated H2B ubiquitylation in *S. cerevisiae*. Deubiquitylation of this ubiquitylated H2B is mediated by DUBm; this modification is necessary for the subsequent recruitment of Ctk1p^104^, which phosphorylates Ser2 in the RNA polymerase II CTD (Fig. 2b). pSer2 crucially supports the transition to productive elongation through its regulatory role in promoter-proximal pausing, suggesting that DUBm regulates the release of paused RNA polymerase II. A more recent study showed that SAGA binding is highly correlated with the pausing site located at the 5′ end of the genes in *D. melanogaster*^105^. Another study found that the level of H2Bub1 was decreased by depletion of dDsk2, that this effect coincided with a disruption in RNA polymerase II pausing at several developmental target genes and that the dDsk2-depletion-induced downregulation of H2Bub1 can be suppressed by the codepletion of Nonstop^106^.

### The SAGA DUBm in mRNA export and surveillance

In addition to regulating transcription activation and elongation through histone modifications, SAGA has been shown to be important for the proper export of mRNAs. SAGA shares its DUBm subunit, Sus1p, with the TREX-2 complex;^71^ this functional and physical interaction between the two complexes enables SAGA to function in maintaining genome stability and coordinating mRNA export^93,107^ (Fig. 2c). Interestingly, several studies have suggested that the proteasome may also contribute to mRNA export through SAGA and TREX-2. The evolutionarily conserved proteasomal 19S RP subunit, Sem1p, which is another TREX-2 complex subunit^108^, is involved in the recruitment and deubiquitinating activity of SAGA^109^. Notably, the 19S RP can nonproteolytically alter the biochemical properties of SAGA to promote gene activation^110^. Rpt2p, which is an ATPase subunit of 19S RP, has been shown to induce the separation of a functional DUBm from SAGA by physically interacting with Sgf73p of DUBm. This separation is essential for the localization of Mex67-Mtr2 and the TREX-2 complex to the transcriptional machinery and thus successful mRNA export^93^. Furthermore, a recent study showed that Sgf73p contributes to mRNP surveillance and the Mex67-mediated noncanonical mRNA export pathway under stress conditions^111^. This study also suggested the possibility of an independent role for Sgf73p separate from the deubiquitylation process. More specifically, the study showed that Sgf73p is the sole factor required for growth restoration of the mRNA export defective mutant *yra1-1*, although the DUBm separated from SAGA upon Rpt2p-Sgf73p interaction has functional deubiquitylation activity in vivo^93^. Nevertheless, H2B ubiquitylation itself seems to be essential, as H2Bub1 and H2Bub1-dependent Swd2p ubiquitylation are required for Mex67p recruitment and export-competent mRNP biogenesis^112^. Last, Sgf73p has been suggested to promote the translocation of the transcription site to the nuclear periphery, which is a phenomenon known as “gene gating”^74,113,114^. This action is believed to allow efficient export of newly transcribed mRNAs. In the future, researchers should study the molecular details underlying the ability of DUBm-mediated deubiquitylation to regulate mRNA biogenesis and assess whether DUBm or Sgf73p plays a role irrespective of deubiquitylation activity.

### SAGA and human disease

Owing to the importance of SAGA in proper gene expression, malfunction of SAGA subunits is likely to cause developmental defects or diseases^115,116^. During development, gene expression must be tightly regulated at each stage to ensure that restricted sets of required genes are activated at specific times while other sets remain silent. A defect in SAGA can disable sequential gene activation, impairing normal development. For example, Drosophila requires Gcn5p for normal metamorphosis, oogenesis, and cell proliferation in imaginal tissues^103,117^. In mouse, *GCN5* depletion causes embryonic lethality^118^, *GCN5* hypomorphic mutants show a defect in neural crest closure^119^, and conditional depletion of Gcn5 results in a decrease in brain size^120^. Although *PCAF*-null mice exhibit normal embryonic development, the double deletion of *GCN5* and *PCAF* causes a more severe phenotype than that observed following depletion of *GCN5* alone^121^. This outcome suggests that GCN5 and PCAF have both redundant and distinct roles. A recent study in zebrafish revealed that knocking down *GCN5* or *PCAF* disturbs cardiac and limb development^122^. Not confined to the HAT catalytic subunit, SAGA itself seems to be important for proper development, as mice harboring a hypomorphic mutant allele of *SUPT20* (the mouse homolog of yeast Spt20p) displayed defects in axial skeletal development^123^. SAGA is also important for maintaining the stemness of stem cells. The well-known target of GCN5 acetyltransferase is c-Myc, which is a pluripotency factor known to be important in the early reprogramming phase of pluripotency induction^124^. Thus, the activation of c-Myc by GCN5 overexpression and enhanced SAGA recruitment facilitates reprogramming. Similarly, a *GCN5* overexpression gain-of-function mutation has been identified as a source of c-Myc-driven cancers in various tissues^125–130^. SAGA activates the transcription of c-Myc and is recruited to c-Myc-targeted genes through interactions between c-Myc and SAGA subunits (e.g., TRRAP, STAF65γ, and KAT2A)^131–133^. Since many c-Myc-dependent genes are related to cell proliferation and cell growth^134^, this interaction may further accelerate malignancies.

Another remarkable subunit of SAGA that appears to be highly engaged in cancer is USP22, which is the catalytic subunit of DUBm. USP22 was initially identified in microarray screens as a member of an 11-gene “death-from cancer” signature that can be used to predict tumor recurrence, metastasis, highly aggressive tumors, and poor prognosis for people with one of several types of cancers^135,136^. More recent studies have shown that USP22 is not only a marker of aggressive tumors but also a tumor inducing factor. USP22 plays an essential role in regulating gene activation and cell growth and in maintaining genome integrity. Usp22 itself regulates transcription through its deubiquitylation activity and is also involved in regulating several TFs, including the androgen receptor^137^, the oncogene c-MYC^88^, and the tumor suppressor p53^138^. USP22 was also shown to act as a key factor in cell cycle progression through the G1 phase by controlling CCND1 ubiquitylation in non-small-cell lung cancer^139^. Further, USP22 has been linked to the maintenance of genome integrity by modulating the stability of TRF1, which regulates the length of a telomere and whose misregulation causes chromosomal abnormalities and cell death^140^. Interestingly, the depletion of GCN5 does not alter TRF1 mRNA expression, and the expression of catalytically inactive GCN5 does not affect TRF1 protein expression, but the loss of GCN5 alters the TRF1 protein level because it impairs the associations of USP22 and ATXN7L3 with SAGA. Overall, the evidence indicates that overexpression of USP22 in cancer leads to the abnormal activation of several pathways involved in cell survival and tumorigenesis. Thus, the development of new cancer therapies targeting USP22 may yield promising results.

In addition to its contribution to cancer development, the DUBm subunit ATXN7 is closely related to neurodegenerative disease. An expansion of a highly conserved polyQ motif in the amino-terminal region of ATXN7 causes spinocerebellar ataxia type 7 (SCA7)^141^. Several studies have indicated that polyQ-expanded ATXN7 (polyQ-ATXN7) is incorporated into SAGA^142,143^ and affects its function. Nuclear inclusions formed by misfolded polyQ-ATXN7 have been found in the cell nuclei of SCA7 patients; these nuclear inclusions also contain other proteins, including the SAGA components GCN5^144^, USP22^145^, and ATXN7L3^146^. PolyQ-ATXN7 has been suggested to further compromise the integrity of SAGA, as it was found to alter the stability of subunits such as GCN5 and STAF36^142^. Although another study failed to observe a change in the level of GCN5 in the polyQ-ATXN7-containing SAGA, the levels of other key SAGA subunits (i.e., Ada2, Ada3, Taf12, and Spt3) were reduced^147^. PolyQ-ATXN7 has also been shown to decrease the HAT activity of SAGA in some studies^142,147^, while others have found that polyQ-ATXN7 increases H2B ubiquitylation without decreasing H3 acetylation^144,146^. Further research is needed to resolve this discrepancy. Although GCN5 depletion may also contribute to the severity of SCA7 phenotypes, it is not sufficient to drive SCA7 in a mouse model^148^. As the loss of GCN5 affects the stability of DUBm^140^, this observation may suggest that the HAT-independent activity of SAGA plays a regulatory role in SCA7.

## Conclusion

SAGA was initially found to be important for both transcriptional activation and elongation, and more recent studies have shown that the complex is also important for mRNA export, especially through the DUBm. SAGA has also been reported to contribute to the biogenesis, quality control, and export of mRNPs. Collectively, these findings show that the SAGA complex intricately coordinates gene expression from the activation step through elongation and export. It seems likely that the structural organization of its functional modules allows SAGA to execute comprehensive and dynamic regulation of gene expression.

It was long believed that SAGA and TFIID selectively regulate the expression of distinct gene groups^149^, but recent reports have suggested that the SAGA complex plays a more general role in gene expression^150^. Nonetheless, the highly regulated genes, whose expression has been suggested to be more dependent on SAGA than TFIID, tend to have TSS-upstream nucleosomes with higher occupancy and less well-defined positioning compared to those of housekeeping genes. For the proper expression of these genes, chromatin-level regulation is required to resolve the competition between the nucleosome and the TF. Indeed, several studies have demonstrated interactions between SAGA and chromatin remodelers. For example, the SAGA-acetylated nucleosome is displaced by SWI/SNF, and Chd1p^151^ is thought to physically associate with SAGA^152^. These studies seem to suggest that SAGA and these chromatin remodelers cooperate to regulate transcription. Additional studies are needed to examine the possible functional links between SAGA and other chromatin remodelers critical for regulating the chromatin structure of the promoter. Understanding the selectivity of specific remodelers and the detailed mechanisms underlying their coordinated action will help researchers elucidate how SAGA functions to fine-tune gene expression.

Based on the current evidence, it is tempting to speculate that mammalian SAGA, ADA, and ATAC function to acetylate histone H3 (and possibly H4) at specific genomic locations through the distinct recruitment mechanisms and/or subunit contexts of each complex. Genome-wide studies to investigate the global localization of each complex in a mammalian system will provide the fundamental information needed to verify this hypothesis. Furthermore, comparative genomics of the localization of yeast SAGA and mammalian HAT complexes may provide insight into how the roles of SAGA have been modified throughout evolution.

To improve our understanding of the functions of SAGA, researchers should seek to resolve the complete structure of SAGA. The structures of some modules have been published, but the literature lacks their functional configurations when bound to chromatin as part of the whole complex and in different cellular contexts. In addition, the process and mechanisms through which SAGA is assembled from individual subunits to an intact 19-protein complex require much more research. Information on whether each subunit undergoes a conformational change upon binding to one another, upon binding of a TF, and/or during the assembly of the PIC will provide invaluable insights into the overall function of SAGA.

## References

[1] Workman JL, Kingston RE (1998). Alteration of nucleosome structure as a mechanism of transcriptional regulation. Annu. Rev. Biochem..

[2] Carrozza MJ, Utley RT, Workman JL, Cote J (2003). The diverse functions of histone acetyltransferase complexes. Trends Genet..

[3] Brownell JE (1996). Tetrahymena histone acetyltransferase A: a homolog to yeast Gcn5p linking histone acetylation to gene activation. Cell.

[4] Grant PA (1997). Yeast Gcn5 functions in two multisubunit complexes to acetylate nucleosomal histones: characterization of an Ada complex and the SAGA (Spt/Ada) complex. Genes Dev..

[5] Martinez E, Kundu TK, Fu J, Roeder RG (1998). A human SPT3-TAFII31-GCN5-L acetylase complex distinct from transcription factor IID. J. Biol. Chem..

[6] Brand M, Yamamoto K, Staub A, Tora L (1999). Identification of TATA-binding protein-free TAFII-containing complex subunits suggests a role in nucleosome acetylation and signal transduction. J. Biol. Chem..

[7] Huisinga KL, Pugh BF (2004). A genome-wide housekeeping role for TFIID and a highly regulated stress-related role for SAGA in *Saccharomyces cerevisiae*. Mol. Cell.

[8] Kremer SB, Gross DS (2009). SAGA and Rpd3 chromatin modification complexes dynamically regulate heat shock gene structure and expression. J. Biol. Chem..

[9] Vinayachandran, V. et al. Widespread and precise reprogramming of yeast protein-genome interactions in response to heat shock. *Genome Res.* **28**, 357–366 (2018). 10.1101/gr.226761.117 PMC5848614 29444801

[10] Ghosh S, Pugh BF (2011). Sequential recruitment of SAGA and TFIID in a genomic response to DNA damage in *Saccharomyces cerevisiae*. Mol. Cell. Biol..

[11] Nagy Z (2009). The human SPT20-containing SAGA complex plays a direct role in the regulation of endoplasmic reticulum stress-induced genes. Mol. Cell. Biol..

[12] Gavin AC (2002). Functional organization of the yeast proteome by systematic analysis of protein complexes. Nature.

[13] Ho Y (2002). Systematic identification of protein complexes in *Saccharomyces cerevisiae* by mass spectrometry. Nature.

[14] Henry KW (2003). Transcriptional activation via sequential histone H2B ubiquitylation and deubiquitylation, mediated by SAGA-associated Ubp8. Genes Dev..

[15] Daniel JA (2004). Deubiquitination of histone H2B by a yeast acetyltransferase complex regulates transcription. J. Biol. Chem..

[16] Fischer T (2004). Yeast centrin Cdc31 is linked to the nuclear mRNA export machinery. Nat. Cell Biol..

[17] Jani D, Valkov E, Stewart M (2014). Structural basis for binding the TREX2 complex to nuclear pores, GAL1 localisation and mRNA export. Nucleic Acids Res..

[18] Koutelou E, Hirsch CL, Dent SYR (2010). Multiple faces of the SAGA complex. Curr. Opin. Cell Biol..

[19] Helmlinger D, Tora L (2017). Sharing the SAGA. Trends Biochem. Sci..

[20] Roberts SM, Winston F (1997). Essential functional interactions of SAGA, a *Saccharomyces cerevisiae* complex of Spt, Ada, and Gcn5 proteins, with the Snf/Swi and Srb/mediator complexes. Genetics.

[21] Wu P-YJ, Ruhlmann C, Winston F, Schultz P (2004). Molecular Architecture of the *S. cerevisiae* SAGA Complex. Mol. Cell.

[22] Brand M (2001). UV-damaged DNA-binding protein in the TFTC complex links DNA damage recognition to nucleosome acetylation. EMBO J..

[23] Martinez E (2001). Human STAGA complex is a chromatin-acetylating transcription coactivator that interacts with pre-mRNA splicing and DNA damage-binding factors in vivo. Mol. Cell. Biol..

[24] Stegeman R (2016). The spliceosomal protein SF3B5 is a novel component of *Drosophila* SAGA that functions in gene expression independent of splicing. J. Mol. Biol..

[25] Kuo M-H (1996). Transcription-linked acetylation by Gcn5p of histones H3 and H4 at specific lysines. Nature.

[26] Sun J (2018). Structural basis for activation of SAGA histone acetyltransferase Gcn5 by partner subunit Ada2. Proc. Natl Acad. Sci. USA.

[27] Jacobson S, Pillus L (2009). The SAGA subunit Ada2 functions in transcriptional silencing. Mol. Cell. Biol..

[28] Nagy Z, Tora L (2007). Distinct GCN5/PCAF-containing complexes function as co-activators and are involved in transcription factor and global histone acetylation. Oncogene.

[29] Linares LK (2007). Intrinsic ubiquitination activity of PCAF controls the stability of the oncoprotein Hdm2. Nat. Cell Biol..

[30] Mazza D (2013). PCAF ubiquitin ligase activity inhibits Hedgehog/Gli1 signaling in p53-dependent response to genotoxic stress. Cell Death Differ..

[31] Eberharter A (1999). The ADA complex is a distinct histone acetyltransferase complex in *Saccharomyces cerevisiae*. Mol. Cell. Biol..

[32] Lee, K. K. et al. Combinatorial depletion analysis to assemble the network architecture of the SAGA and ADA chromatin remodeling complexes. *Mol. Syst. Biol.* **7**10.1038/msb.2011.40 (2011). 10.1038/msb.2011.40 PMC3159981 21734642

[33] Soffers JHM (2019). Characterization of a metazoan ADA acetyltransferase complex. Nucleic Acids Res..

[34] Suganuma T (2008). ATAC is a double histone acetyltransferase complex that stimulates nucleosome sliding. Nat. Struct. Mol. Biol..

[35] Nagy Z (2010). The metazoan ATAC and SAGA coactivator HAT complexes regulate different sets of inducible target genes. Cell. Mol. Life Sci..

[36] Guelman S (2006). Host cell factor and an uncharacterized SANT domain protein are stable components of ATAC, a novel dAda2A/dGcn5-containing histone acetyltransferase complex in *Drosophila*. Mol. Cell. Biol..

[37] Kusch T, Guelman S, Abmayr SM, Workman JL (2003). Two Drosophila Ada2 homologues function in different multiprotein complexes. Mol. Cell Biol..

[38] Muratoglu S (2003). Two different Drosophila ADA2 homologues are present in distinct GCN5 histone acetyltransferase-containing complexes. Mol. Cell Biol..

[39] Guelman S (2009). The double-histone-acetyltransferase complex ATAC is essential for mammalian development. Mol. Cell Biol..

[40] Riss A (2015). Subunits of ADA-two-A-containing (ATAC) or Spt-Ada-Gcn5-acetyltrasferase (SAGA) Coactivator Complexes Enhance the Acetyltransferase Activity of GCN5. J. Biol. Chem..

[41] Orpinell M (2010). The ATAC acetyl transferase complex controls mitotic progression by targeting non-histone substrates. EMBO J..

[42] Spedale G, Timmers HT, Pijnappel WW (2012). ATAC-king the complexity of SAGA during evolution. Genes Dev..

[43] Mi W (2018). The ZZ-type zinc finger of ZZZ3 modulates the ATAC complex-mediated histone acetylation and gene activation. Nat. Commun..

[44] Krebs AR, Karmodiya K, Lindahl-Allen M, Struhl K, Tora L (2011). SAGA and ATAC histone acetyl transferase complexes regulate distinct sets of genes and ATAC defines a class of p300-independent enhancers. Mol. Cell.

[45] Bonnet J (2014). The SAGA coactivator complex acts on the whole transcribed genome and is required for RNA polymerase II transcription. Genes Dev..

[46] Gangloff YG, Romier C, Thuault S, Werten S, Davidson I (2001). The histone fold is a key structural motif of transcription factor TFIID. Trends Biochem. Sci..

[47] Ogryzko VV (1998). Histone-like TAFs within the PCAF histone acetylase complex. Cell.

[48] Sterner DE (1999). Functional organization of the yeast SAGA complex: distinct components involved in structural integrity, nucleosome acetylation, and TATA-binding protein interaction. Mol. Cell Biol..

[49] Wang H (2020). Structure of the transcription coactivator SAGA. Nature.

[50] Elías-Villalobos A, Toullec D, Faux C, Séveno M, Helmlinger D (2019). Chaperone-mediated ordered assembly of the SAGA and NuA4 transcription co-activator complexes in yeast. Nat. Commun..

[51] Belotserkovskaya R (2000). Inhibition of TATA-binding protein function by SAGA subunits Spt3 and Spt8 at Gcn4-activated promoters. Mol. Cell. Biol..

[52] Wu PY, Winston F (2002). Analysis of Spt7 function in the *Saccharomyces cerevisiae* SAGA coactivator complex. Mol. Cell. Biol..

[53] Papai G (2020). Structure of SAGA and mechanism of TBP deposition on gene promoters. Nature.

[54] Eisenmann DM, Chapon C, Roberts SM, Dollard C, Winston F (1994). The *Saccharomyces cerevisiae* SPT8 gene encodes a very acidic protein that is functionally related to SPT3 and TATA-binding protein. Genetics.

[55] McMahon SB, Van Buskirk HA, Dugan KA, Copeland TD, Cole MD (1998). The novel ATM-related protein TRRAP is an essential cofactor for the c-Myc and E2F oncoproteins. Cell.

[56] Saleh A (1998). Tra1p is a component of the yeast Ada.Spt transcriptional regulatory complexes. J. Biol. Chem..

[57] Hoffman KS (2016). *Saccharomyces cerevisiae* Tti2 regulates PIKK proteins and stress response. G3.

[58] Berg MD, Genereaux J, Karagiannis J, Brandl CJ (2018). The pseudokinase domain of *Saccharomyces cerevisiae* Tra1 is required for nuclear localization and incorporation into the SAGA and NuA4 complexes. G3.

[59] Knutson B, Hahn S (2011). Domains of Tra1 important for activator recruitment and transcription coactivator functions of SAGA and NuA4 complexes. Mol. Cell. Biol..

[60] Sharov G (2017). Structure of the transcription activator target Tra1 within the chromatin modifying complex SAGA. Nat. Commun..

[61] Allard S (1999). NuA4, an essential transcription adaptor/histone H4 acetyltransferase complex containing Esa1p and the ATM-related cofactor Tra1p. EMBO J..

[62] Cheung ACM, Díaz-Santín LM (2019). Share and share alike: the role of Tra1 from the SAGA and NuA4 coactivator complexes. Transcription.

[63] Helmlinger D (2011). Tra1 has specific regulatory roles, rather than global functions, within the SAGA co-activator complex. EMBO J..

[64] Liu G (2019). Architecture of *Saccharomyces cerevisiae* SAGA complex. Cell Discov..

[65] Wang X, Ahmad S, Zhang Z, Côté J, Cai G (2018). Architecture of the *Saccharomyces cerevisiae* NuA4/TIP60 complex. Nat. Commun..

[66] Bruzzone MJ, Grünberg S, Kubik S, Zentner GE, Shore D (2018). Distinct patterns of histone acetyltransferase and Mediator deployment at yeast protein-coding genes. Genes Dev..

[67] Weake VM, Workman JL (2008). Histone ubiquitination: triggering gene activity. Mol. Cell.

[68] Ingvarsdottir K (2005). H2B ubiquitin protease Ubp8 and Sgf11 constitute a discrete functional module within the *Saccharomyces cerevisiae* SAGA complex. Mol. Cell. Biol..

[69] Lee KK, Florens L, Swanson SK, Washburn MP, Workman JL (2005). The deubiquitylation activity of Ubp8 is dependent upon Scf11 and its association with the SAGA complex. Mol. Cell. Biol..

[70] Powell DW (2004). Cluster analysis of mass spectrometry data reveals a novel component of SAGA. Mol. Cell. Biol..

[71] Rodriguez-Navarro S (2004). Sus1, a functional component of the SAGA histone acetylase complex and the nuclear pore-associated mRNA export machinery. Cell.

[72] Köhler A (2006). The mRNA export factor Sus1 is involved in Spt/Ada/Gcn5 acetyltransferase-mediated H2B deubiquitinylation through its interaction with Ubp8 and Sgf11. Mol. Biol. Cell.

[73] Sanders SL, Jennings J, Canutescu A, Link AJ, Weil PA (2002). Proteomics of the eukaryotic transcription machinery: identification of proteins associated with components of yeast TFIID by multidimensional mass spectrometry. Mol. Cell. Biol..

[74] Köhler A, Schneider M, Cabal GG, Nehrbass U, Hurt E (2008). Yeast Ataxin-7 links histone deubiquitination with gene gating and mRNA export. Nat. Cell Biol..

[75] Lee KK, Swanson SK, Florens L, Washburn MP, Workman JL (2009). Yeast Sgf73/Ataxin-7 serves to anchor the deubiquitination module into both SAGA and Slik(SALSA) HAT complexes. Epigenet. Chromatin.

[76] Köhler A, Zimmerman E, Schneider M, Hurt E, Zheng N (2010). Structural basis for assembly and activation of the heterotetrameric SAGA histone H2B deubiquitinase module. Cell.

[77] Samara NL (2010). Structural insights into the assembly and function of the SAGA deubiquitinating module. Science.

[78] Durand A, Bonnet J, Fournier M, Chavant V, Schultz P (2014). Mapping the deubiquitination module within the SAGA complex. Structure.

[79] Han Y, Luo J, Ranish J, Hahn S (2014). Architecture of the *Saccharomyces cerevisiae* SAGA transcription coactivator complex. EMBO J..

[80] Morgan MT (2016). Structural basis for histone H2B deubiquitination by the SAGA DUB module. Science.

[81] Koehler C (2014). DNA Binding by Sgf11 protein affects histone H2B deubiquitination by Spt-Ada-Gcn5-Acetyltransferase (SAGA). J. Biol. Chem..

[82] Bonnet J (2010). The structural plasticity of SCA7 domains defines their differential nucleosome‐binding properties. EMBO Rep..

[83] Gallego LD (2016). Structural mechanism for the recognition and ubiquitination of a single nucleosome residue by Rad6-Bre1. Proc. Natl Acad. Sci. USA.

[84] Kurshakova MM (2007). SAGA and a novel *Drosophila* export complex anchor efficient transcription and mRNA export to NPC. EMBO J..

[85] Weake VM (2008). SAGA-mediated H2B deubiquitination controls the development of neuronal connectivity in the *Drosophila* visual system. EMBO J..

[86] Mohan RD (2014). Loss of *Drosophila* Ataxin-7, a SAGA subunit, reduces H2B ubiquitination and leads to neural and retinal degeneration. Genes Dev..

[87] Helmlinger D (2004). Ataxin-7 is a subunit of GCN5 histone acetyltransferase-containing complexes. Hum. Mol. Genet..

[88] Zhang X-Y (2008). The putative cancer stem cell marker USP22 is a subunit of the human SAGA complex required for activated transcription and cell-cycle progression. Mol. Cell.

[89] Zhao Y (2008). A TFTC/STAGA module mediates histone H2A and H2B deubiquitination, coactivates nuclear receptors, and counteracts heterochromatin silencing. Mol. Cell.

[90] Lang G (2011). The tightly controlled deubiquitination activity of the human SAGA complex differentially modifies distinct gene regulatory elements. Mol. Cell. Biol..

[91] Vermeulen M (2010). Quantitative interaction proteomics and genome-wide profiling of epigenetic histone marks and their readers. Cell.

[92] Li W (2016). Cytoplasmic ATXN7L3B interferes with nuclear functions of the SAGA deubiquitinase module. Mol. Cell. Biol..

[93] Lim S, Kwak J, Kim M, Lee D (2013). Separation of a functional deubiquitylating module from the SAGA complex by the proteasome regulatory particle. Nat. Commun..

[94] Belotserkovskaya R (2003). FACT facilitates transcription-dependent nucleosome alteration. Science.

[95] Pavri R (2006). Histone H2B monoubiquitination functions cooperatively with FACT to regulate elongation by RNA polymerase II. Cell.

[96] Fleming AB, Kao C-F, Hillyer C, Pikaart M, Osley MA (2008). H2B ubiquitylation plays a role in nucleosome dynamics during transcription elongation. Mol. Cell.

[97] Chandrasekharan MB, Huang F, Sun Z-W (2009). Ubiquitination of histone H2B regulates chromatin dynamics by enhancing nucleosome stability. Proc. Natl Acad. Sci. USA.

[98] Batta K, Zhang Z, Yen K, Goffman DB, Pugh BF (2011). Genome-wide function of H2B ubiquitylation in promoter and genic regions. Genes Dev..

[99] Fierz B (2011). Histone H2B ubiquitylation disrupts local and higher-order chromatin compaction. Nat. Chem. Biol..

[100] Machida, S., Sekine, S., Nishiyama, Y., Horikoshi, N. & Kurumizaka, H. Structural and biochemical analyses of monoubiquitinated human histones H2B and H4. **6**, 160090, 10.1098/rsob.160090 (2016). 10.1098/rsob.160090 PMC4929944 27335322

[101] Krajewski WA, Li J, Dou Y (2018). Effects of histone H2B ubiquitylation on the nucleosome structure and dynamics. Nucleic Acids Res..

[102] Shukla A, Stanojevic N, Duan Z, Sen P, Bhaumik SR (2006). Ubp8p, a histone deubiquitinase whose association with SAGA is mediated by Sgf11p, differentially regulates lysine 4 methylation of histone H3 in vivo. Mol. Cell. Biol..

[103] Li X (2017). Enzymatic modules of the SAGA chromatin-modifying complex play distinct roles in *Drosophila* gene expression and development. Genes Dev..

[104] Wyce A (2007). H2B ubiquitylation acts as a barrier to Ctk1 nucleosomal recruitment prior to removal by Ubp8 within a SAGA-related complex. Mol. Cell.

[105] Weake VM (2011). Post-transcription initiation function of the ubiquitous SAGA complex in tissue-specific gene activation. Genes Dev..

[106] Kessler R (2015). dDsk2 regulates H2Bub1 and RNA polymerase II pausing at dHP1c complex target genes. Nat. Commun..

[107] Pascual-Garcia P (2008). Sus1 is recruited to coding regions and functions during transcription elongation in association with SAGA and TREX2. Genes Dev..

[108] Faza MB (2009). Sem1 is a functional component of the nuclear pore complex-associated messenger RNA export machinery. J. Cell Biol..

[109] Garcia-Oliver E (2013). A novel role for Sem1 and TREX-2 in transcription involves their impact on recruitment and H2B deubiquitylation activity of SAGA. Nucleic Acids Res..

[110] Lee D (2005). The proteasome regulatory particle alters the SAGA coactivator to enhance its interactions with transcriptional activators. Cell.

[111] Kim M, Choi Y, Kim H, Lee D (2019). SAGA DUBm-mediated surveillance regulates prompt export of stress-inducible transcripts for proteostasis. Nat. Commun..

[112] Vitaliano-Prunier A (2012). H2B ubiquitylation controls the formation of export-competent mRNP. Mol. Cell.

[113] Cabal GG (2006). SAGA interacting factors confine sub-diffusion of transcribed genes to the nuclear envelope. Nature.

[114] Luthra R (2007). Actively transcribed GAL genes can be physically linked to the nuclear pore by the SAGA chromatin modifying complex. J. Biol. Chem..

[115] Wang L, Dent SYR (2014). Functions of SAGA in development and disease. Epigenomics.

[116] Trisciuoglio D, Di Martile M, Del Bufalo D (2018). Emerging role of histone acetyltransferase in stem cells and cancer. Stem Cells Int..

[117] Carre C, Szymczak D, Pidoux J, Antoniewski C (2005). The histone H3 acetylase dGcn5 is a key player in *Drosophila* melanogaster metamorphosis. Mol. Cell Biol..

[118] Xu W (2000). Loss of Gcn5l2 leads to increased apoptosis and mesodermal defects during mouse development. Nat. Genet..

[119] Lin W (2008). Proper expression of the Gcn5 histone acetyltransferase is required for neural tube closure in mouse embryos. Dev. Dyn..

[120] Martinez-Cerdeno V (2012). N-Myc and GCN5 regulate significantly overlapping transcriptional programs in neural stem cells. PLoS ONE.

[121] Yamauchi T (2000). Distinct but overlapping roles of histone acetylase PCAF and of the closely related PCAF-B/GCN5 in mouse embryogenesis. Proc. Natl Acad. Sci. USA.

[122] Ghosh TK (2018). Acetylation of TBX5 by KAT2B and KAT2A regulates heart and limb development. J. Mol. Cell. Cardiol..

[123] Warrier S, Nuwayhid S, Sabatino JA, Sugrue KF, Zohn IE (2017). Supt20 is required for development of the axial skeleton. Dev. Biol..

[124] Hirsch CL (2015). Myc and SAGA rewire an alternative splicing network during early somatic cell reprogramming. Genes Dev..

[125] Liu K (2015). GCN5 potentiates glioma proliferation and invasion via STAT3 and AKT signaling pathways. Int. J. Mol. Sci..

[126] Majaz S (2016). Histone acetyl transferase GCN5 promotes human hepatocellular carcinoma progression by enhancing AIB1 expression. Cell Biosci..

[127] Zhao L, Pang A, Li Y (2018). Function of GCN5 in the TGF-beta1-induced epithelial-to-mesenchymal transition in breast cancer. Oncol. Lett..

[128] Farria AT, Mustachio LM, Akdemir ZHC, Dent SYR (2019). GCN5 HAT inhibition reduces human Burkitt lymphoma cell survival through reduction of MYC target gene expression and impeding BCR signaling pathways. Oncotarget.

[129] Mustachio LM, Roszik J, Farria AT, Guerra K, Dent SY (2019). Repression of GCN5 expression or activity attenuates c-MYC expression in non-small cell lung cancer. Am. J. Cancer Res..

[130] Mustachio, L. M., Roszik, J., Farria, A. & Dent, S. Y. R. Targeting the SAGA and ATAC transcriptional coactivator complexes in MYC-driven cancers. *Cancer Res.*10.1158/0008-5472.Can-19-3652 (2020). 10.1158/0008-5472.CAN-19-3652 PMC7231639 32094302

[131] McMahon SB, Wood MA, Cole MD (2000). The essential cofactor TRRAP recruits the histone acetyltransferase hGCN5 to c-Myc. Mol. Cell Biol..

[132] Flinn EM (2002). Recruitment of Gcn5-containing complexes during c-Myc-dependent gene activation. Structure and function aspects. J. Biol. Chem..

[133] Liu X, Tesfai J, Evrard YA, Dent SY, Martinez E (2003). c-Myc transformation domain recruits the human STAGA complex and requires TRRAP and GCN5 acetylase activity for transcription activation. J. Biol. Chem..

[134] Mannava S (2008). Direct role of nucleotide metabolism in C-MYC-dependent proliferation of melanoma cells. Cell Cycle.

[135] Glinsky GV, Berezovska O, Glinskii AB (2005). Microarray analysis identifies a death-from-cancer signature predicting therapy failure in patients with multiple types of cancer. J. Clin. Investig..

[136] Glinsky GV (2005). Death-from-cancer signatures and stem cell contribution to metastatic cancer. Cell Cycle.

[137] Schrecengost RS (2014). USP22 regulates oncogenic signaling pathways to drive lethal cancer progression. Cancer Res..

[138] Lin Z (2012). USP22 antagonizes p53 transcriptional activation by deubiquitinating Sirt1 to suppress cell apoptosis and is required for mouse embryonic development. Mol. Cell.

[139] Gennaro VJ (2018). Control of CCND1 ubiquitylation by the catalytic SAGA subunit USP22 is essential for cell cycle progression through G1 in cancer cells. Proc. Natl Acad. Sci. USA.

[140] Atanassov BS (2009). Gcn5 and SAGA regulate shelterin protein turnover and telomere maintenance. Mol. Cell.

[141] Niewiadomska-Cimicka A, Trottier Y (2019). Molecular targets and therapeutic strategies in spinocerebellar ataxia type 7. Neurotherapeutics.

[142] Palhan VB (2005). Polyglutamine-expanded ataxin-7 inhibits STAGA histone acetyltransferase activity to produce retinal degeneration. Proc. Natl Acad. Sci. USA.

[143] Helmlinger, D., Hardy, S., Eberlin, A., Devys, D. & Tora, L. Both normal and polyglutamine- expanded ataxin-7 are components of TFTC-type GCN5 histone acetyltransferase- containing complexes. *Biochem. Soc. Symp*. 155–163, 10.1042/bss0730155 (2006). 10.1042/bss0730155 16626296

[144] McCullough SD (2012). Reelin is a target of polyglutamine expanded ataxin-7 in human spinocerebellar ataxia type 7 (SCA7) astrocytes. Proc. Natl Acad. Sci. USA.

[145] Yang H (2015). Aggregation of polyglutamine-expanded ataxin 7 protein specifically sequesters ubiquitin-specific protease 22 and deteriorates its deubiquitinating function in the Spt-Ada-Gcn5-Acetyltransferase (SAGA) Complex. J. Biol. Chem..

[146] Lan XJ (2015). Poly(Q) expansions in ATXN7 affect solubility but not activity of the SAGA deubiquitinating module. Mol. Cell. Biol..

[147] McMahon SJ, Pray-Grant MG, Schieltz D, Yates JR, Grant PA (2005). Polyglutamine-expanded spinocerebellar ataxia-7 protein disrupts normal SAGA and SLIK histone acetyltransferase activity. Proc. Natl Acad. Sci. USA.

[148] Chen YC (2012). Gcn5 loss-of-function accelerates cerebellar and retinal degeneration in a SCA7 mouse model. Hum. Mol. Genet..

[149] Fischer V, Schumacher K, Tora L, Devys D (2019). Global role for coactivator complexes in RNA polymerase II transcription. Transcription.

[150] Baptista T (2018). SAGA is a general cofactor for RNA polymerase II transcription. Mol. Cell.

[151] Chandy M, Gutierrez JL, Prochasson P, Workman JL (2006). SWI/SNF displaces SAGA-acetylated nucleosomes. Eukaryot. Cell.

[152] Pray-Grant MG, Daniel JA, Schieltz D, Yates JR, Grant PA (2005). Chd1 chromodomain links histone H3 methylation with SAGA- and SLIK-dependent acetylation. Nature.

